# The role of interest in the transmission of social values

**DOI:** 10.3389/fpsyg.2013.00349

**Published:** 2013-06-17

**Authors:** Fabrice Clément, Daniel Dukes

**Affiliations:** ^1^Cognitive Science Centre, University of NeuchâtelNeuchâtel, Switzerland; ^2^Swiss Center for Affective Sciences, University of GenevaGeneva, Switzerland

**Keywords:** appraisal theory, interest, social appraisal, social values, social learning

## Abstract

The environment is so rich with information that our cognitive system would be overloaded without a way to evaluate what is relevant for our needs and goals. Appraisal theory has shown how emotions, by “tagging” the environment with differential values, enable the attribution of our attentional resources to what is most relevant in any given circumstances. Most often, however, the different cues triggering the allocation of attention are thought of as purely individualistic, like physiological needs or past encounters with certain stimuli. This approach is perfectly appropriate for objects, organisms or events that, by their intrinsic properties, affect the organism's well being. But for humans, many aspects of the environment are culturally or temporally dependent: a soccer game may be highly relevant to some, but not at all to others. This paper contributes to a better understanding of the processes by which different elements of our social environment acquire value through our socialization process. We recruit different concepts proposed by developmental psychologists to shed some light on this social acquisition of relevance. The notion of “joint attention,” for example, is particularly important to understand how we are sensitive to the other's focus of attention. Similarly, the term “social referencing” has been used to describe the process of taking into account the affective reaction to a given stimuli, in order to direct our behavior. At the core of this process, called “social appraisal” by Manstead, we propose that a specific emotion plays a major role: interest. Someone else's expression of interest, which seems to be detectable from a very early age, is extremely useful in gauging what is worthy of attention among stimuli that are not inherently interesting. The paper highlights how external sources of information (the life experiences of community members) indicate what is relevant, thus giving access to the social values of that group.

## Introduction

In the long empiricist tradition that characterizes the Western conception of humankind, our inner experience of the world results from successive perceptual contacts with our biological, physical and social environment. In this context, it is hard from a developmental perspective not to think of a baby's mental life as “one great blooming, buzzing confusion,” (James, 1890/1981, p. 462). How could it be otherwise in a world of objects and events that overlap and coincide in so many ways? Many of the recent breakthroughs in developmental psychology have been aimed at understanding the relative placidity of babies confronted with a plethora of information that they in fact, master impressively quickly. Everything happens as if babies were naturally equipped for such “cognitive digestion,” either because they can rely on some evolutionary modules to make sense of the information due to some core knowledge—naive physics, biology, psychology, or even sociology (Baillargeon, 1994; Wellman and Woolley, 1990; Hirschfeld and Gelman, 1994; Spelke, 1994; Xu and Carey, 1996), or because they rely on powerful “pattern detectors” which enable them to detect correlations and forge hypotheses about the structure of the world (Gopnik, 2010).

These cognitive predispositions, however, are just part of the solution. Even if we imagine that babies are equipped to process in specific ways, how can they assess which information should be processed at any given time? Without a system enabling them to prioritize how to distribute their cognitive powers, the risk of behavioral paralysis is too high.

Appraisal theory provides a possible answer since it describes how not all stimuli are equal to our cognitive system, and how our emotions play the role of “radar antennae scanning the environment,” (Scherer, 1994, p. 230). This relative saliency in our environment can be due to the fact that, given our organism's needs, certain stimuli have a high biological significance and are therefore automatically prioritized by the attentional system (Brosch et al., 2007). For instance, when hungry, the value of food is very high and priority is given to any process leading to the satisfying of this biological need. Other factors are more personal and depend on the individual's history: a given odor or taste for instance, can mobilize a person's attention because it reminds her of a special time with her grandmother. Finally, certain elements of our environment are socially relevant because they are considered as important for the members of the individual's reference group. In certain communities for example, a soccer World Cup Final will thus be lived as though it is a religious ritual, whereas in others, it will be ignored. For all these values, the amygdala seems to play an essential role in filtering what is relevant to the organism, and an event is relevant if it can “significantly influence (positively or negatively) the attainment of his or her goals, the satisfaction of his or her needs, the maintenance of his or her own well-being, and the well-being of his or her species” (Sander et al., 2003, p. 311).

In this paper, we will focus on social relevance and, more specifically, on how socio-cultural values pass from socialized to newly arrived social members, i.e., babies. After a brief summary of the component process model (CPM) proposed by Klaus Scherer to describe how we appraise incoming stimuli, we will concentrate on a dimension of the model that has not been given much attention: normative significance. By specifying the nature of this check, we will propose that recent attempts to describe social appraisal are particularly relevant to this topic. More specifically, we will show how the emotion of interest can be essential for the baby to discern what is valued in his or her social environment when it is displayed by significant others. To specify the competences required for “using” expressed interest to detect what is socially relevant, we will revisit three important notions that underlie the way infants can be influenced by others in their development: joint attention, social referencing and social appraisal. Consequences for the study of the role of interest will then be discussed.

## The social side of appraisal

Nowadays, the CPM is one of the most complex, empirically supported and heuristic models of emotion processing (Scherer, 1984, 2001; Ellsworth and Scherer, 2003). In this model, emotions are regarded as psychological episodes that have a felt character and are evaluative of particular objects (Deonna and Teroni, 2012). Its central idea is that emotions play a key role in the way our brains scan the environment and prepare our organism for action (Leventhal and Scherer, 1987). This evaluation is performed by a series of different checks that occur in a sequential order—Stimulus Evaluation Checks (SEC). Everything happens as if the emotional processes respond to the following questions: “(a) How relevant is this event for me? Does it directly affect me or my social reference group? (relevance); (b) What are the implications or consequences of this event and how do they affect my well-being and my immediate or long-term goals? (implications); (c) How well can I cope with or adjust to these consequences? (coping potential); (d) What is the significance of this event for my self-concept and for social norms and values? (normative significance)” (Scherer, 2009, p. 1309). These checks, that often occur automatically, unconsciously and effortlessly, are supposed to follow this order.

In this model, relevance detection plays an essential role because it is considered to be a first selective filter that a stimulus or event needs to pass through in order to merit further processing (Sander et al., 2005, p. 322). However, the role played by social factors at this stage remains unclear. On the one hand, the significance of the stimulus or the event with respect to social norms and values is clearly relegated to one of the final evaluation checks. It consists in checking the compatibility with external standards: social norms, values, beliefs about justice, or moral principles (Scherer, 2009, p. 1313). By definition, it requires high-level and comprehensive information, and even “comparison with high-level propositional representation” (Sander et al., 2005, p. 322). On the other hand, the social nature of the relevance process is acknowledged: relevance is about how a given event affects oneself or one's *social reference group* (Sander et al., 2005, p. 319). Indeed, the role of social context in appraisal has been highlighted by recent work underlining, in the CPM perspective, the role played by the amygdala for relevance. Sander et al. (2003) suggested that the human amygdala works as a “relevance detector” and is activated in presence of social signals such as gaze direction, intentions, group adherence, trustworthiness and facial familiarity. Other works have highlighted the role played by the amygdala when individuals have to evaluate trustworthiness in their social exchanges (Adolphs et al., 1998; Winston et al., 2002; Todorov and Engell, 2008). Given the rapidity of these relevance detection processes driven by the amygdala (Vuilleumier, 2005; Brosch et al., 2008), one can therefore conclude that at least a part of what is social in the CMP does not need reflexive and/or propositional processes.

If the processing of another person's facial or bodily expressions triggers the amygdala, especially when the person is looking directly at you (Conty and Grèzes, 2012), it is unlikely that these intersubjective situations are the only way that appraisal processes are influenced by the “social.” Indeed, relevance is evaluated according to the significance events have for what is valued by the organism. Certain stimuli have high biological significance and are automatically prioritized by the attention system (Brosch et al., 2007), while others trigger specific evaluations of the environment as a result of personal needs and values (Ellsworth and Scherer, 2003). Social relevance aims precisely at understanding how these personal values are shaped during the development of a person. Admittedly, values do not emerge in a social void. On the contrary, children most likely develop a certain number of their personal preferences and values as a result of the contact they have with their social referents (parents, friends, teachers, coach, etc.). Once this transmission period ends, specific parts of their environment will more or less automatically trigger their attention and interest. For a child born into a family of cyclists, for instance, a champion like Eddy Merckx would be immediately detected in a crowd and this episode would be remembered forever, while on the contrary, the cyclists' hero would stay unnoticed by a family of soccer players. Therefore, depending on the “attentional” priorities of their social milieu, an individual's environment tends to be colored by different “social lenses” that will render certain elements of the world as valuable and worthy of attention, at the same time sentencing the others to indifference and invisibility.

Therefore, it seems very important (even from a psychological point of view) to understand how values, inherent in a given form of life (Clément, 1996), pervade our appraisal system, to the point that it can influence the very beginning of the SEC. As the following section will show, developmental psychology appears to offer some indications.

## Attending to others

In many senses, the human species is fundamentally a social species. As the anthropologist Clifford Geertz wrote: “human behavior is so loosely determined by intrinsic sources of information that extrinsic sources are so vital” (Geertz, 1973/1993, p. 93). Even if not credulous, children learn most of what they know via others' testimony (Clément et al., 2004; Clément, 2010). Moreover, the human species is fundamentally altricial: offspring are highly dependent on others for a very long period of time. It is therefore not very surprising that other people's appraisal systems can influence one's own evaluation of events and stimuli.

From a cognitive perspective, newborns seem to be “prewired” for attending to human-like faces (Johnson et al., 1991; Heron-Delaney et al., 2011). This preference may well be due to the fact that there is an evolutionary advantage for babies in treating other human faces as particularly relevant to making sense of their surroundings, given their richness as a source of information. Furthermore, from an early age, babies are increasingly able to follow someone else's gaze (Scaife and Bruner, 1975). For instance, 2–5-day-old newborns can discriminate between direct and averted gaze, and 4-month-old infants' brain activity shows specific neural activity when presented with faces with direct (as opposed to averted) eye gaze (Farroni et al., 2002). A few months later, evidence begins to emerge that infants start looking at the world via others' perspectives. At 12 months, infants are able to detect selective attention when an adult looks at several things but attends only to some part of them (Tomasello and Haberl, 2003). This capacity, called *joint attention*, plays a crucial role in development, notably in language learning. Indeed, it is thanks to joint attention that caregivers and infants can establish what is being referenced, and learn that certain sounds match with objects, persons or events in the shared environment (Tomasello, 2003). From a perceptual point of view, it is therefore very likely that infants are prone to select their objects of attention, at least partially, by aligning their own attention with others' attention.

Beside the ability to take into account others' objects of interest and to be driven to be attentive to the same objects, babies around that age (the end of their first year) start to move around on their own, and they gain new ways of feeding their appetite for exploration. Facing all kinds of new objects, they have ever more opportunities to create mischief. Fortunately perhaps, it is also at this age that they start to rely more on their caregivers' cues (facial expressions, body language and tone of voice) to appraise ambiguous and new events (Klinnert et al., 1986). As Feinman and Lewis (1983) put it, caregivers serve from now on not only as a base of security but also as a base of information. Such social information gathering “is rooted in the ability to appreciate that another individual can function as a conduit for information about the world” (Baldwin and Moses, 1996, p. 1917). In the famous visual cliff experiment, infants dared to crawl over a simulated cliff when the mothers expressed joy or interest (Sorce et al., 1985). This ability that the infant has to disambiguate the emotional meaning of objects in the environment by actively seeking out emotional information from significant others (Hertenstein and Campos, 2004) has been called *social referencing*. It is important to highlight the fact that, contrary to joint attention where infants' focus of attention is driven by another's gaze direction, the object of concern for social referencing pre-exists in the infant's conscious field. In a way, the child has already evaluated the object as relevant, and the social information she obtains is essentially used to modulate her behavior toward that object. We propose therefore, a slightly less inclusive definition than Feinman and Lewis (1983, p. 878), who define social referencing as the use of one's perception of someone else's interpretations of a situation to form one's own understanding of that situation. We agree more readily with Pelaez et al.'s (2012) definition of social referencing as “a behavior chain in which the presence of an ambiguous object or event signals the gaze shift of an infant toward another person, typically the mother, whose facial, vocal, and gestural expressions may then serve as discriminative stimuli for a subsequent approach response” (p.23). We therefore endorse the view that social referencing directs behavior, rather than forms an understanding.

In contrast to the aforementioned cases, there are situations where the focus of a given object or event does not pre-exist the social interaction, or when the evaluation of the object itself is modified by the nature of the social information. Children, for instance, can be intrigued by the way adults are captivated and excited by a soccer game on television (Demers et al., 2013); in those families, we can expect children to be sensitive to future soccer related events. On the contrary, the appraisal that underlies an activity like stuffing oneself with ice cream can be modified by a strong and negative emotional parental reaction. Even occurrent emotional reactions triggered by an individual appraisal, for instance, bursting out laughing when seeing an old man stumbling in a bus, can be re-evaluated once the emotional reactions of the other, disapproving witnesses are taken into consideration (Jakobs et al., 1997). To refer to these cases where the value of events or objects are modified by the observation of other people's emotional reactions, Manstead and his colleagues (Manstead and Fischer, 2001; Evers et al., 2005) have proposed the concept of *social appraisal*. One of Manstead's objectives is to highlight the fact that most appraisal theorists tended to favor research that focuses on relatively socially isolated individuals, and on values that are essentially independent from the socio-cultural environment. In contrast, social appraisal highlights the fact that “the behaviors, thoughts or feelings of one or more other persons are often appraised in addition to the appraisal of the event *per se*” (Manstead and Fischer, 2001, p. 222).

Social appraisal can be expected to play a considerable part in a child's socialization, given that there are many events and objects in our social environment that are not relevant in terms of their intrinsic properties, and the fact our social environment is full of objects that arouse considerable interest for certain groups of people, but not to others.

It is only via others' appraisal that the relevance of a particular artistic form, sport, hobby, political engagement, or environmental consciousness becomes salient for the children. However, while there is an abundance of developmental research on joint attention and social referencing, the role of social appraisal has not really been identified in infancy. Compared to social referencing, where others' emotions seem to play a regulatory role in the expression of a behavior by encouraging or discouraging the on-going action, it is most likely that social appraisal necessitates a much finer understanding of the expressed emotion. To play the role of relevancy detectors, others' faces have to be “read” by the children: only a rather subtle interpretation of others' appraisal can help them to detect if an object or event is worthy of attention, on a scale going from “abhorrent” to “highly desirable.” In this context, we hypothesize that certain expressed emotions play an essential role by “tagging” certain stimuli with a given emotional valence for the children. Such a transmission of values can be intentional and explicit: parents, for instance, may find that is very important to transmit their love of the arts, or the virtue of politeness, to their heirs. In these cases, parents may resort to what Gergerly and Csibra call *natural pedagogy* with ostensive communication to indicate new and relevant information (Gergely and Csibra, 2006; Csibra and Gergely, 2009). It has been shown in such pedagogical contexts that mothers adapt their voice and speech when talking to young children, speaking “motherese” (Snow, 1972). More generally, adults also modify their movements when interacting with infants such that their actions simultaneously enhance infant's attention and highlight meaningful units within the flow of motion (Brand et al., 2002). More specifically, when mothers show objects to young children, relative to showing them to other adults, their actions are notably characterized by closer proximity to the partner, greater enthusiasm, a larger range of motion, greater repetitiveness, longer gazes, more turn-taking and greater simplification (Brand et al., 2007). However, we argue that not all social transmissions of values rely on such ostensive cues. By observing others, children (and adults) can detect what captures their attention or, on the contrary, what they disregard: an expression of awe or an expression full of scorn, even expressed by an anonymous by-stander, can still be very socially relevant.

For this third-party influence in the ontogenesis of social relevance, we suppose that certain expressed emotions will play an essential role, notably disgust, contempt, and interest. We will focus here on interest because (1) it is an emotion that has not yet been extensively studied, (2) it should indicate to an observer what another person appraises as being “worthy of interest”, i.e., as *relevant*. Interest is therefore an emotion of crucial importance for social appraisal.

## The role of interest

Given the scope of this paper, we cannot discuss here all the aspects of interest (but see Silvia, 2006). Briefly, interest is the emotion associated with curiosity, exploration, and information seeking (Tomkins, 1962; Berlyne, 1966; Izard, 2009). According to Silvia, interest as a felt emotion consists of appraisals of novelty (factors related to the unfamiliarity and complexity of an object or event) and appraisals of coping potential (the ability to understand the new object or event) (Silvia, 2005). Its function is to motivate seeking behaviors, learning and exploration (Panksepp, 2005; Silvia, 2008). One of the important questions in studying interest concerns the existence of a specific expression of interest. This aspect is especially important given our problematic: social appraisal could not take place without cues that enable children to detect others' interest.

The expression of interest has apparently no place among the most renowned and widely used basic emotion stimuli that Ekman considered as universal: happiness, sadness, anger, fear, disgust, and surprise (Ekman and Friesen, 1971). Actually, it appears from later research that Ekman had considered including both “interest” and “contempt” in the series, but presumably he was unable to find suitable static photographs (Ekman, 1992, 1993). However, even if “momentary expressions” are particularly efficient from an evolutionary perspective, Ekman did not deny the important role that “extended expressions” might play (Ekman, 1993). Interest seems precisely to be one of these extended emotions and it is therefore not surprising that even adults cannot recognize static stimuli of interest. This was first identified in a 1964 study where interest was one of eight expressions presented to participants (neutrality, surprise, distress, enjoyment, fear, anger, disgust, shame, and interest) and where interest was only more frequently recognized than surprise (Tomkins and Mc Carter, 1964). Interestingly, Tomkins and Mc Carter report that the actors they had hired to pose the expressions complained particularly about how difficult it was to pose “interest.” We had a similar difficulty with the actors that were hired for a study we are currently conducting with adults, as if playing a static interest, contrary to a dynamic one, was impossible. In our experiment, participants are asked to watch pictures or movies and to recognize the staged emotions based on Ekman's basic expressions, including a neutral/calm expression and the expression of interest. Static headshots are not well recognized for interest, particularly when compared to six-second films of dynamic headshots and dynamic whole body shots (Dukes et al., in preparation). Similar results were found in another study in which four positive emotions (pride, pleasure, joy, and interest) were compared (Mortillaro et al., 2011). Facial expressions of each emotion were taken from the Geneva Multimodal Emotion Portrayal corpus in which each actor was asked to express each emotion several times [see Bänzinger et al. (2012) for details]. Representative facial expressions were then coded using the Facial Action Coding System (FACS). While the four positive emotions could not be differentiated on the basis of the presence or absence of particular Actions Units (AU), they could be differentiated in terms of their temporal dynamics—the sequence and timing of the unfolding expression [see Krumhuber et al. (2013) for a review of the dynamics of facially expressed affect].

From the perspective of studying the role of expressed interest in the ontogenesis of social appraisal, it will therefore be essential to expose infants to dynamic stimuli. The other important point to be assessed is the “contagion” of the interest. In other words, does the observation of someone being interested by an object cause children to also appraise this object with interest? One of the dimensions is *behavioral*. As interest is the emotion that underlies curiosity, seeking and exploratory behaviors are expected toward an object that has been considered with interest by a third-party. A more subtle behavioral dimension is eye gaze pattern: by using an eye-tracker, it should be possible to detect whether an object of someone's interest triggers more curiosity and becomes more visually explored by the participant. Another dimension is the transmission of the emotion itself: when an infant sees a person being interested in something, will he/she start being interested in the same object? Such an inquiry is rather complicated because one has to identify on babies' faces the signs of interest. Actually, several early studies have described the facial expression of interest in babies. One such study argued that babies as young as 9 months were able to express interest (Izard, 1980), while another study suggested that infants may facially signal emotions, including interest (Oster, 1978). A further study described several facial movements as indicative of an expression of interest, such as brows raised, brows knit, eyes widened and rounded, eyes squinted, cheeks raised, mouth opened and relaxed, tongue moved, lips pursed [(Izard, 1979); Izard, as cited in Langsdorf et al. (1983)]. Using these indications, Langsdorf et al. (1983) showed that facial expressions of interest predicted the time that the infants spent viewing human or inanimate objects while Izard et al. (1995) show that expressions of interest were morphologically stable between the ages of 2.5 and 9 months. Another important facet of the expression of interest appears to be the “body stilling and facial sobering” (Camras et al., 2002) or “freezing” (Scherer et al., 2004) that characterizes a reaction to a novel stimulus: the whole body and facial expression remains motionless for a moment after the stimulus becomes known. It has been argued that freezing is a normal reaction to an ambiguous situation, as the person is unsure how to react, and that this is more likely to occur in very young infants who “do not yet have the necessary cognitive mechanisms (nor the stored experiences) to conclusively appraise highly unusual events and to prepare appropriate action tendencies” (Scherer et al., 2004, p. 399). It is as if the organism is “buying time” to disambiguate the situation before reacting. Of course, the importance that freezing may play in an expression of interest shows again why interest might be more recognizable when presented dynamically.

By putting together what we currently know about (a) the way interest is expressed and (b) the different cues indicating that infants are experiencing the emotion of interest (behavior, eye gaze patterns, and felt interest), it is possible to conceive of studies that seek to understand when and how children are able to take into account the attentional parsing of the environment performed by their caregivers. Given that joint attention is assumed to emerge around the age of 12 months (Carpenter et al., 1998; Moll and Tomasello, 2004), we suspect that this ability emerges during the second year. Furthermore, as children have been shown to learn a lot about their social environment by observation alone (Rogoff, 2003), it is likely that this third-party appraisal does not require any ostensive signals from the adults in order to be accomplished.

## Conclusion

The main objective of this paper was to consider more carefully the role played by others in the determination of what is relevant in our surrounding. Apart from some very basic biological values, most of the objects, events, and phenomena we consider as worthy of investment in time, energy or resources, we in fact inherit from our social and cultural environment. It seems therefore important to study, in an appraisal theory perspective, the last step of the SEC proposed by Klaus Scherer: normative significance.

Our feeling is that, via social information gathered by children from very early on, norms and values are so deeply embedded in the appraisal process that even the first evaluation check—how relevant is this event for me?—is marked by the social history of the individuals.

When scanning the environment, some objects or events seem more salient than others. These objects/events are often more salient because they are relevant to the individual's goals. We have shown the importance of an individual's life experience in the detection of what is relevant and therefore their “choice” of object about which they will appraise. This does not mean that this experience has not been tainted by numerous encounters with significant others who shared, explicitly or not, what they considered as interesting, appalling, or insignificant. But, at a given time, all these life experiences can act as an *internal* source of information when appraising an event. In other situations, the importance of the third person's perspective can be brought to the forefront in the appraisal process: in this case, it is some *external* source of information (the life experience of another person) that will influence the appraisal. Clearly, this third person directs her attention as a consequence of her own life experiences and values, which again were elaborated in contact with others. When these multiple social appraisals happen in a relatively interconnected circle, nothing less than a culture is transmitted and perpetuated.

To study the very beginning of this cultural process and to prepare the ground for experimental studies with infants, we proposed a conceptual gradation in the way children take advantage of social information in the early stages of their development. From a *perceptual* point of view, babies are sensitive to others' direction of gaze. At the end of their first year, they can detect others' selective attention and join their own attention to those of their caregivers—*joint attention*. From a *behavioral* point of view, they can, at around the same period, actively seek emotional information from significant others to modulate their own behavior—*social referencing*. Eventually, most likely in the second year, infants are able to take into consideration an emotion expressed by another person to appraise an event, object or person—*social appraisal*.

The emotion of *interest* appears to be particularly relevant for studying the onset and development of social appraisal by children. In expressing interest, adults offer important cues about what is salient for them in their environment. We hypothesize that every expression of interest that children can detect on an adult's face and body, enables them to “tag” their environment with different levels of saliency. Social appraisal plays therefore a crucial role for children: it enables them to enter a given society by gaining access to the values that are essential to the members of their reference groups.

### Conflict of interest statement

The authors declare that the research was conducted in the absence of any commercial or financial relationships that could be construed as a potential conflict of interest.
